# Supplementary material for: Using Deep Learning Models of Gene Regulation to Guide Drug Prioritization
Source: bioRxiv. 2026 May 14:2026.05.11.724354. Preprint. [Version 1] doi: 10.64898/2026.05.11.724354 (PMC13192863; doi:10.64898/2026.05.11.724354)
Supplement: Supplement 1 [file media-1.pdf]

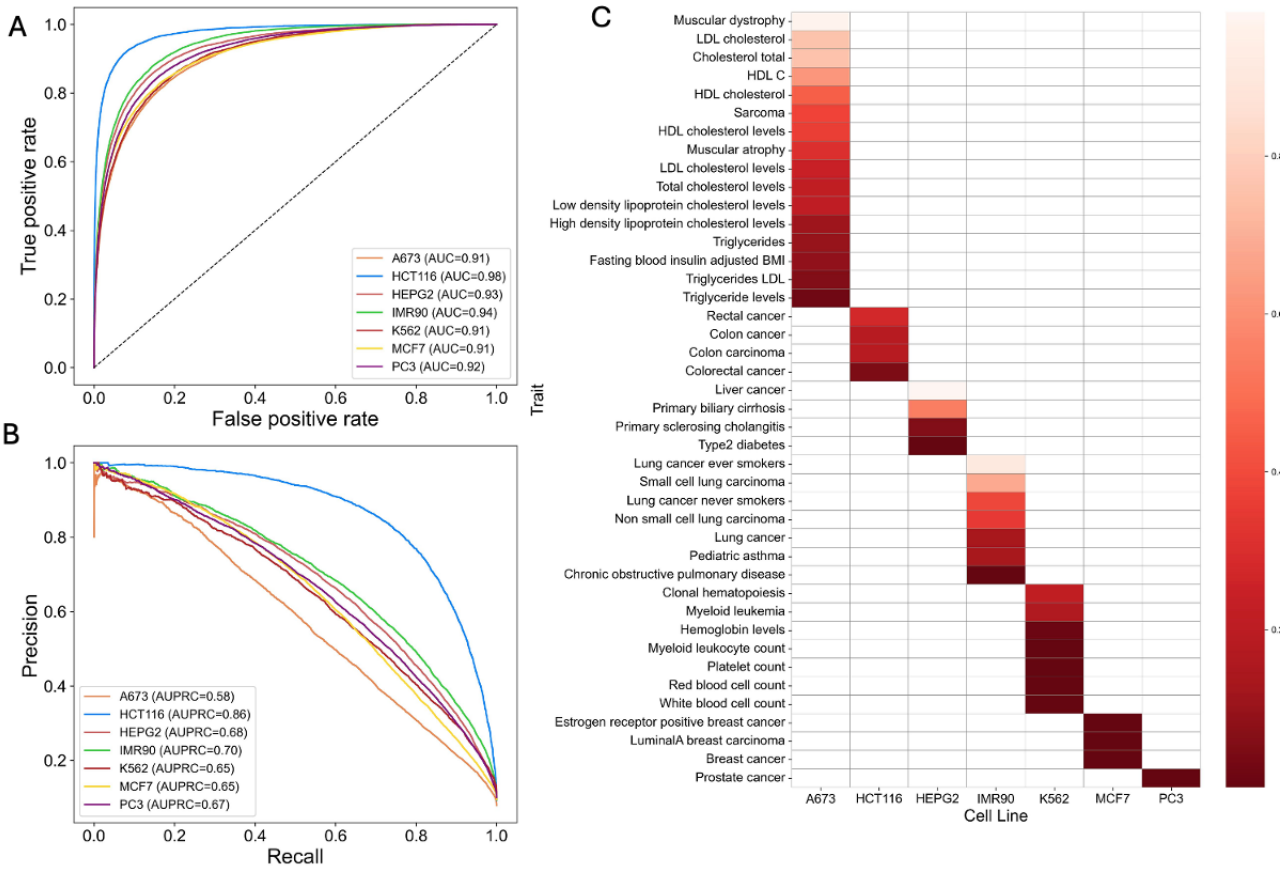

Figure S1 Cell line-specific enhancer model performance and GWAS heritability enrichment. (A) Enhancer model performance (AUC) on the test set from a representative fold of chromosome-level cross-validation. (B) Enhancer model performance (AUPRC) on the test set from a representative fold of chromosome-level cross-validation. (C) GWAS heritability enrichment across cell line-specific enhancer annotations.

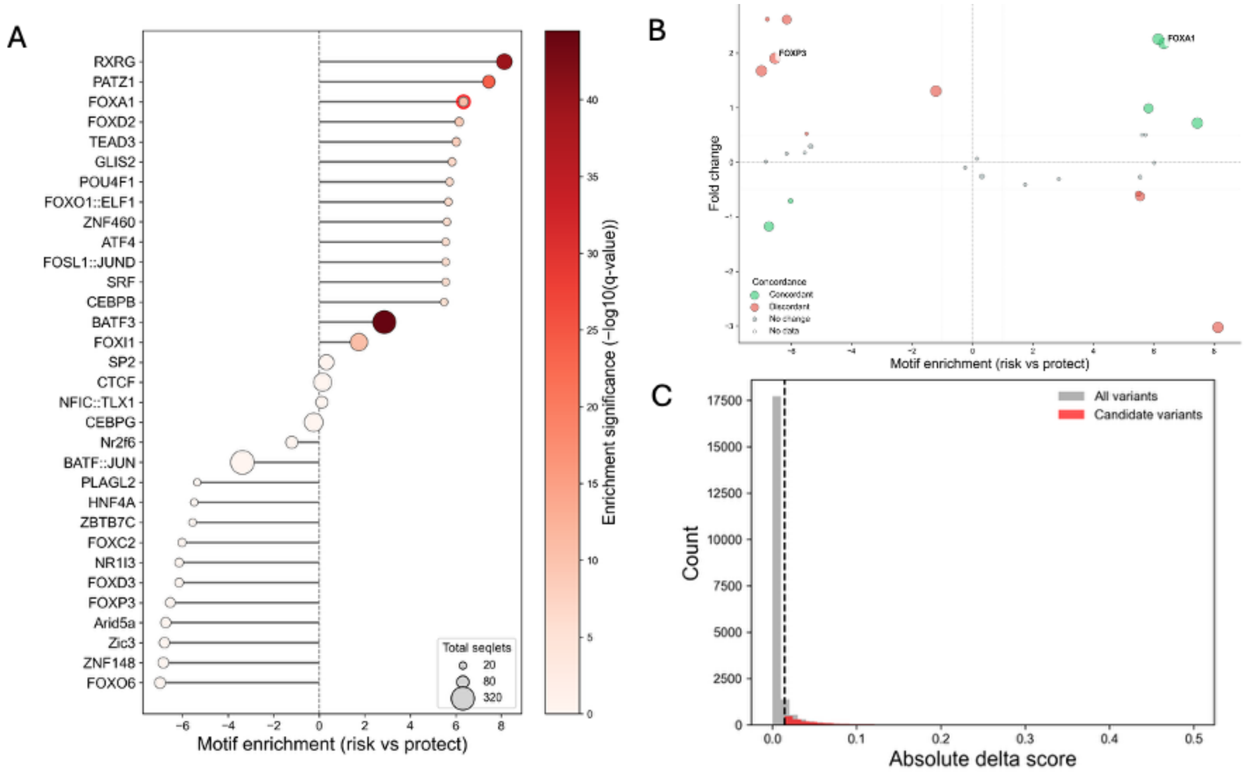

Figure S2. Allele-dependent motif enrichment and tumor expression concordance in breast cancer. (A) Transcription factor motifs identified from breast cancer GWAS variants using TREDNet enhancer models and attribution-based TF-MoDISco analysis, ranked by $\log_2$ risk/protective enrichment. Dot size indicates seqlet support; color represents $-\log_{10}$(q-value). (B) Motif enrichment (x-axis; $\log_2$ risk/protective ratio) versus tumor differential expression in TCGA-BRCA (y-axis; $\log_2$ fold change). Concordant TFs (green) show agreement between risk allele enrichment and tumor upregulation. FOXA1 and FOXD2 exhibit strong concordance. Point size reflects $-\log_{10}$(FDR). Dashed lines indicate classification thresholds. (C) Distribution of absolute allele-dependent enhancer prediction differences (|Δscore|) across all variants (gray) and prioritized candidates (red). The dashed vertical line marks the 90th percentile cutoff. Motif enrichment values were computed as $\log_2((n\_risk + 0.5)/(n\_protect + 0.5))$, applying a pseudocount of 0.5 (Haldane-Anscombe correction) to avoid undefined values when the protective allele seqlet count was zero.

Table S1 Summary of TF motif enrichment and tumor expression. Log_2 enrichment values were computed as $log_2((n\_risk + 0.5) / (n\_protect + 0.5))$ using a pseudocount of 0.5 (Haldane-Anscombe correction). Negative log_{10}(q-values) are reported for statistical significance.

TF motif	N-seqlets protect allele	N-seqlets risk allele	$log2^{enrich}$ (risk vs. protect)	$neglog10^q$	Gene	logFC	FDR
BATF3	38	279	2.86	44.50	BATF3	-0.31	0.00
RXRG	0	140	8.13	39.58	RXRG	-3.03	0.00
PATZ1	0	87	7.45	23.91	PATZ1	0.72	0.00
FOXI1	40	135	1.74	10.61	FOXI1	-0.41	0.10
FOXA1	0	40	6.34	10.50	FOXA1	2.18	0.00
FOXD2	0	35	6.15	9.16	FOXD2	2.26	0.00
TEAD3	0	32	6.02	8.37	TEAD3	-0.01	0.87
GLIS2	0	28	5.83	7.29	GLIS2	0.99	0.00
POU4F1	0	26	5.73	6.77	POU4F1	0.50	0.29
FOXO1::ELF1	0	25	5.67	6.53	-	-	-
ZNF460	0	24	5.61	6.29	ZNF460	0.50	0.98
ATF4	0	23	5.55	6.11	ATF4	-0.27	0.00
FOSL1::JUND	0	23	5.55	6.11	-	-	-
SRF	0	23	5.55	6.11	SRF	-0.63	0.00
CEBPB	0	22	5.49	5.86	CEBPB	-0.58	0.00
SP2	57	71	0.31	0.26	SP2	-0.26	0.00
CTCF	90	100	0.15	0.01	CTCF	0.06	0.11
PLAGL2	20	0	-5.36	0.00	PLAGL2	0.29	0.00
ZBTB7C	23	0	-5.55	0.00	ZBTB7C	0.17	0.24
ZNF148	57	0	-6.85	0.00	ZNF148	0.01	0.90
FOXP3	46	0	-6.54	0.00	FOXP3	1.91	0.00
NR1I3	35	0	-6.15	0.00	NR1I3	0.16	0.09
NFIC::TLX1	37	40	0.11	0.00	-	-	-

HNF4A	22	0	-5.49	0.00	HNF4A	0.52	0.00
FOXO6	63	0	-6.99	0.00	FOXO6	1.67	0.00
FOXD3	35	0	-6.15	0.00	FOXD3	2.61	0.00
FOXC2	32	0	-6.02	0.00	FOXC2	-0.71	0.00
CEBPG	110	93	-0.24	0.00	CEBPG	-0.10	0.07
BATF::JUN	305	29	-3.37	0.00	-	-	-
ARID5A	53	0	-6.74	0.00	ARID5A	-1.18	0.00
NR2F6	56	24	-1.21	0.00	NR2F6	1.30	0.00
ZIC3	55	0	-6.79	0.00	ZIC3	2.62	0.00

Table S2 Enrichment of approved drugs among candidate compounds at varying thresholds. P-values were calculated using Fisher's exact test.

Percentile	N-candidate	N-approve	Enrichment fold	Odds ratio	P value
0.75	157	24	1.10	1.13	0.33
0.8	125	21	1.21	1.26	0.20
0.85	94	18	1.38	1.48	0.09
0.9	63	17	1.95	2.32	0.00
0.95	32	6	1.35	1.44	0.28

Table S3 Compound names corresponding to the column order shown from left to right in Figure 4B, C

Z score based	Spearman based
ixazomib	ixazomib
pitavastatin	fulvestrant
pralatrexate	pitavastatin
fulvestrant	pralatrexate
irinotecan	irinotecan
bortezomib	reserpine
carfilzomib	perampanel
perampanel	pentobarbital
pentobarbital	bortezomib
amsacrine	carfilzomib
reserpine	amsacrine
homosalate	mycophenolate-mofetil
bisacodyl	thiothixene
mycophenolate-mofetil	homosalate
clofarabine	bisacodyl
ingenol-mebutate	ingenol-mebutate
thiothixene	clofarabine
floxuridine	floxuridine
BRD-K78385490	cercosporin
BRD-K69894866	BRD-K78385490
BNTX	BRD-K69894866
cercosporin	BVD-523
BRD-A40431293	BRD-K60870698
NNC-55-0396	BRD-K28366633
BRD-K74316684	NSC-3852
JNJ-26481585	JNJ-26481585
genz-644282	BRD-A40431293
camptothecin	NNC-55-0396
BRD-A49848186	CVF-SUMO-11
NSC-3852	ST-056792
BRD-K28366633	BVT-948
AG-592	EMF-sumo1-39
malonoben	AG-592
BRD-K60870698	VU-0418934-2
AMG-232	BRD-A49848186
BVD-523	BRD-K55722623

VU-0418934-2	BRD-K74305673
RG-7388	BRD-K81795824
BRD-K74305673	BRD-A68065211
BRD-K81795824	malonoben
BRD-K52321331	BRD-K74316684
CVF-SUMO-11	BNTX
BRD-A68065211	BRD-K52321331
ST-056792	BRD-K95285735
PHA-848125	AMG-232
EMF-sumo1-39	camptothecin
BRD-A37735495	BRD-K18724229
BRD-K18724229	deguelin
merck-ketone	PHA-848125
deguelin	BRD-A49680073
BRD-K55722623	hycanthone
BVT-948	merck-ketone
YM-155	brazilin
BRD-A49680073	genz-644282
hycanthone	RG-7388
BRD-K95285735	YM-155
R-547	SB-939
brazilin	R-547
dorsomorphin	dorsomorphin
diphenyleneiodonium	BRD-A37735495
BRD-K00313977	BRD-K00313977
SB-939	diphenyleneiodonium
BRD-K18726304	BRD-K18726304

Table S4 Number and fraction of candidate compounds with positive pathway anti-correlation scores and Spearman correlation (spr) across pathways.

pathway	N-positive	Positive (%)	N-positive(spr)	Positive-spr (%)
G2M CHECKPOINT	63	100	63	100
E2F TARGETS	62	98.4	62	98.4
ESTROGEN RESPONSE LATE	62	98.4	61	96.8
MTORC1 SIGNALING	62	98.4	61	96.8
PI3K AKT MTOR SIGNALING	60	95.2	58	92.1
MYC TARGETS V1	59	93.6	58	92.1
UNFOLDED PROTEIN RESPONSE	57	90.5	52	82.5
ESTROGEN RESPONSE EARLY	44	69.8	48	76.2
